# Protocol for extended-plex immunofluorescence staining of FFPE tissues using a sequential bleach-and-stain approach

**DOI:** 10.1016/j.xpro.2026.104806

**Published:** 2026-08-27

**Authors:** Elias Stahl, Yang Zhang, Jan A. Schlegel, Julia Thiel, Chunguang Liang, Werner Schroth, Meng Dong

**Affiliations:** 1 Dr. Margarete Fischer-Bosch Institute of Clinical Pharmacology and University of Tuebingen, 70376 Stuttgart, Germany; 2 Department of Bioinformatics, Biocenter Am Hubland, University of Würzburg, 97074 Würzburg, Germany; 3 Institute of Immunology, Jena University Hospital, Friedrich-Schiller-University, 07743 Jena, Germany; 4 Comprehensive Cancer Center Central Germany (CCCG), Jena University Hospital, 07743 Jena, Germany

**Keywords:** Cancer, Microscopy, Molecular Biology

## Abstract

Multiplex immunofluorescence enables spatial analysis of complex biological systems, but increasing plex levels raise cost and workflow complexity. Here, we present a protocol for extending tyramide signal amplification (TSA)-based multiplex immunofluorescence from 6 to 16 markers using a sequential bleach-and-stain approach. It covers tissue preparation, panel establishment, sequential staining, fluorophore bleaching, image acquisition and computational image processing including stitching, registration, and overlay. The workflow is applicable to formalin-fixed paraffin-embedded (FFPE) human cancer and lymphoid tissues and is adaptable through the selection of appropriate antibodies.

## Highlights

• Protocol for sequential bleach-and-stain multiplex immunofluorescence of FFPE tissues
• Instructions for multiplex panel establishment using conventional antibodies
• Steps for image stitching, registration, and overlay
• Guidance on automated image processing with a provided Python workflow

## Abstract (editor highlights)

Publisher’s note: Undertaking any experimental protocol requires adherence to local institutional guidelines for laboratory safety and ethics.

## Graphical abstract

**Figure:**
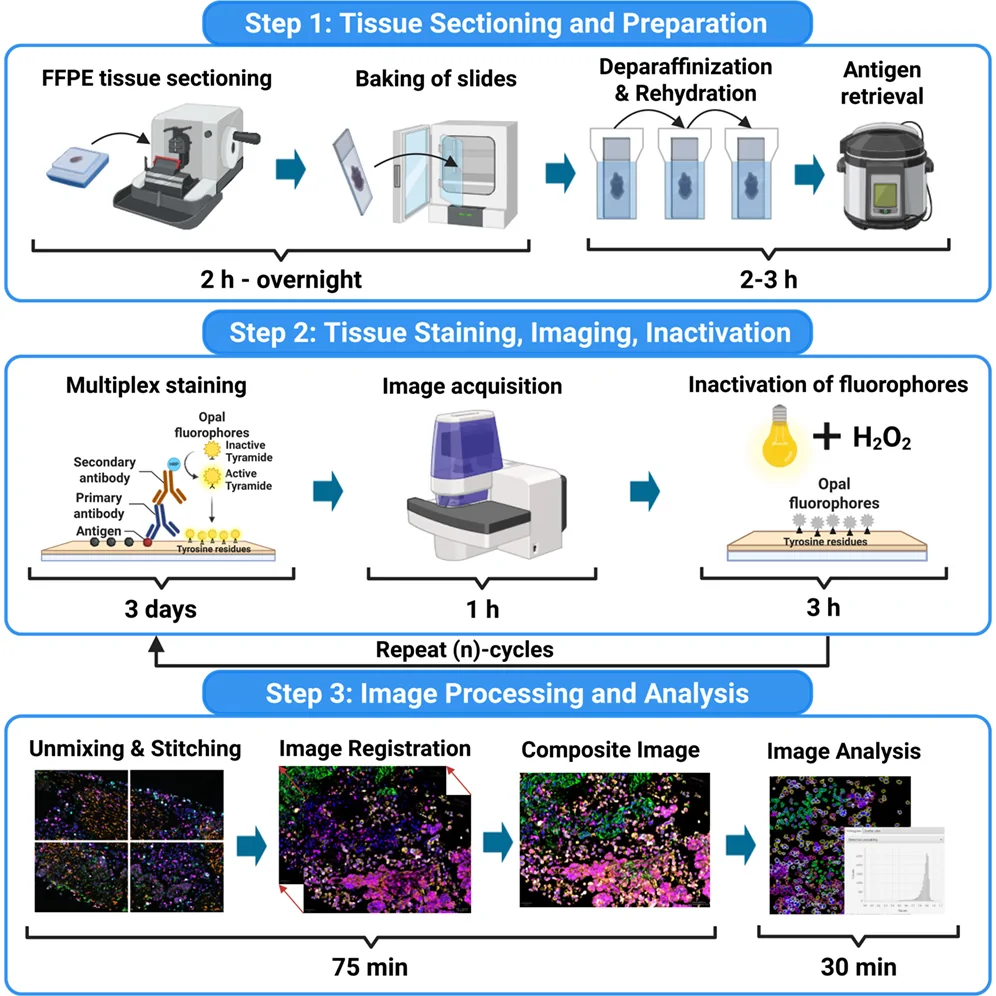

## Teaser

Multiplex immunofluorescence enables spatial analysis of complex biological systems, but increasing plex levels raise cost and workflow complexity. Here, we present a protocol for extending tyramide signal amplification (TSA)-based multiplex immunofluorescence from 6 to 16 markers using a sequential bleach-and-stain approach. The protocol covers FFPE tissue preparation, panel establishment, sequential staining, fluorophore bleaching, image acquisition and computational image processing including stitching, registration, and overlay. The workflow is applicable to FFPE human cancer and lymphoid tissues and is adaptable through the selection of appropriate antibodies.

## Before you begin

This protocol extends the Opal fluorophore staining system via tyramide signal amplification (TSA) from the standard 6-plex setup towards 16-plex levels for targeted spatial analyses using the Akoya imaging platform. Designed for use with conventional antibodies validated for standard immunohistochemistry (IHC), it achieves extended-plex staining through sequential bleach-and-stain cycles without requiring dedicated high-plex spatial technologies. Importantly, this protocol is not intended to replace high-plex spatial technologies capable of detecting more than 60 markers. Instead, it provides an accessible and flexible alternative for targeted multiplex analyses in laboratories using conventional IHC antibodies and TSA-based workflows. Although the protocol is described using Akoya reagents and imaging systems, it is not restricted to proprietary platforms. Similar TSA-based workflows can be implemented using tyramide fluorophores from alternative suppliers, such as Biotium CF dyes, and are compatible with other fluorescence imaging platforms, including confocal microscopy, as demonstrated previously.^1^^,^^2^ These features provide flexibility in both reagent selection and imaging instrumentation while maintaining the overall bleach-and-stain workflow.

A key advantage of this approach is the flexibility of panel design, as Opal fluorophores can be assigned to any validated antibody, allowing panels to be readily expanded or modified without the need for newly generated conjugated antibody sets. In contrast, high-plex platforms based on Oligo-barcoded antibodies, such as the PhenoCycler, require predefined antibody panels, making panel adaptation more expensive, time-consuming and less flexible.

The workflow encompasses tissue sectioning, staining, imaging, and basic analysis using the open-source bioimaging analysis software QuPath, and incorporates a sequential bleach-and-stain strategy to expand multiplexing capacity on the same tissue section. When combined with a Python-based alignment and overlay script, the resulting image contains all markers, providing all necessary information for downstream analysis. This protocol was applied to FFPE tissue sections derived from different soft tissue entities, i.e., ovarian, lung and breast cancers that originate from retrospective studies at our institution. Tissue collection, fixation, and embedding were performed by the institutional pathology department according to standard diagnostic protocols. FFPE tissue blocks were stored at room temperature for up to 5 years prior to sectioning and analysis.

Iterative multiplex immunofluorescence approaches such as tissue-based cyclic immunofluorescence (t-CyCIF)^3^ and iterative bleaching extends multiplexity (IBEX)^4^ have demonstrated the potential of repeated staining and fluorophore inactivation cycles for high-dimensional tissue imaging of up to 60 markers. IBEX employs rapid chemical bleaching using lithium borohydride (LiBH_4_), whereas t-CyCIF uses the conventional fluorophores Alexa Fluor 488, 555/570, and 647 with chemical photobleaching using 4.5% hydrogen peroxide (H_2_O_2_) and 24 mM sodium hydroxide (NaOH) between staining cycles.

In contrast, our workflow was specifically optimized for iterative bleaching of five Opal fluorophores (Opals 480, 520, 570, 620, 690), including Opal 520 and 620, which are not efficiently quenched by LiBH_4_-based protocols such as IBEX.^4^ Furthermore, the chemical photobleaching approach was optimized to use 3% H_2_O_2_, as higher concentrations resulted in tissue detachment and reduced tissue stability during repeated staining cycles, while still ensuring efficient fluorophore inactivation. This enables robust bleach-and-stain cycles within a TSA/Opal-based multiplex immunofluorescence workflow while maintaining compatibility with standard fluorescence imaging platforms.

Image registration and overlay of consecutive staining cycles are critical steps for accurate downstream analysis of multiplex immunofluorescence images. In this workflow, image alignment was performed using a custom Python-based registration pipeline to enable automated stitching, registration and overlay of sequential staining rounds prior to image analysis. Open-source tools such as QuPath^5^ provide image alignment capabilities through the Align extension and represent valuable alternatives for image registration and visualization. However, our pipeline was specifically developed to integrate stitching, registration, and overlay of sequential whole-slide acquisitions within a unified workflow. It provides an automated, user-friendly solution for batch processing and generates a single merged OME-TIFF file containing aligned image layers for downstream visualization and quantitative analysis.

Overall, this protocol provides a comprehensive end-to-end workflow for extending Opal-based multiplex immunofluorescence beyond standard 6-plex applications to higher levels of multiplexing using conventional antibodies established for standard IHC, thus offering a flexible framework for targeted spatial protein analysis. The protocol steps were partially modified from the Opal Multiplex IHC Assay Development Guide from Akoya Biosciences.

**Figure 1:**
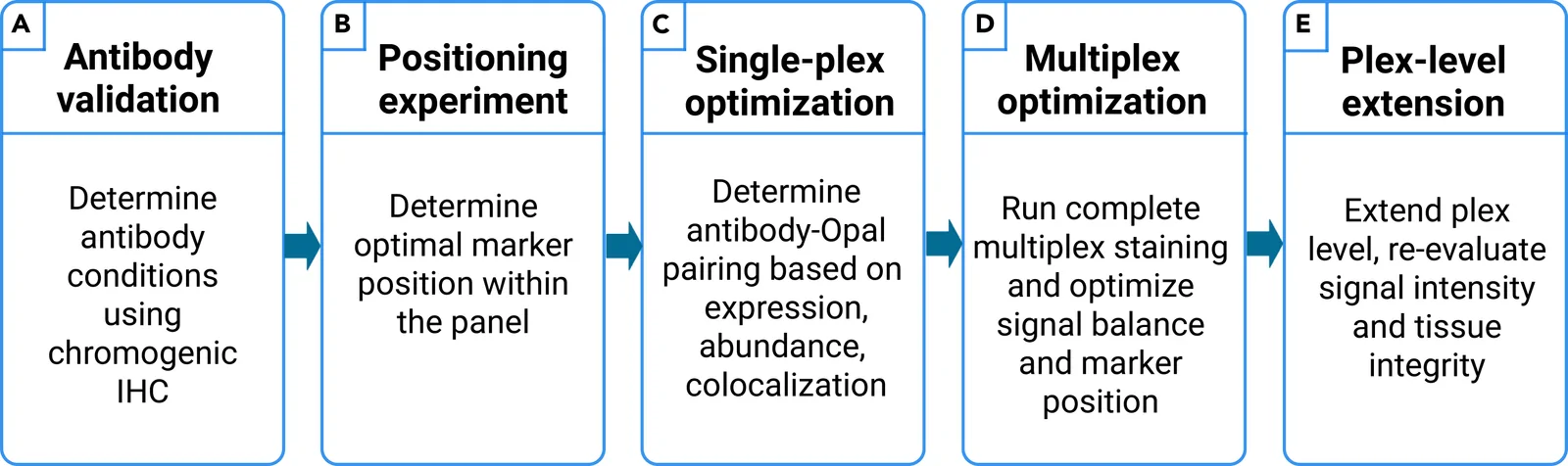
Workflow for multiplex immunofluorescence panel establishment (A) Antibody validation: Antibodies are screened by chromogenic immunohistochemistry (IHC) under varying conditions to determine specificity and optimal signal-to-noise ratio (SNR). (B) Positioning experiment: Antibodies are tested at different panel positions to assess sensitivity of epitopes to repeated heat-induced epitope retrieval (HIER) steps. (C) Single-plex optimization: Validated antibodies are evaluated in Opal single-plex staining to confirm staining quality and to assign Opal fluorophores based on marker abundance, spectral compatibility, and potential co-localization. (D) Multiplex optimization: Full multiplex staining is performed, and final panel adjustments are introduced. (E) Plex-level extension: Iterative bleaching and restaining enable plex extension, with staining quality, signal intensity, and tissue integrity reassessed after each cycle.

The successful implementation of the extended Opal multiplex staining workflow requires several preparatory steps to ensure specificity, reproducibility, and optimal signal quality characteristics with details published elsewhere.^6^^,^^7^ Important steps are discussed here (Figure 1):

1. Antibody validation.
  a. Validate each antibody for specificity and optimal staining conditions prior to multiplex staining (Figure 2A).
    ***Note:*** Use antibody datasheet recommendations as a starting point. Optimize staining conditions using chromogenic immunohistochemistry (IHC) by varying heat-induced epitope retrieval (HIER) conditions (buffer pH 6 or 9), antibody dilution, and incubation time to determine the optimal signal-to-noise ratio (SNR).

**Figure 2:**
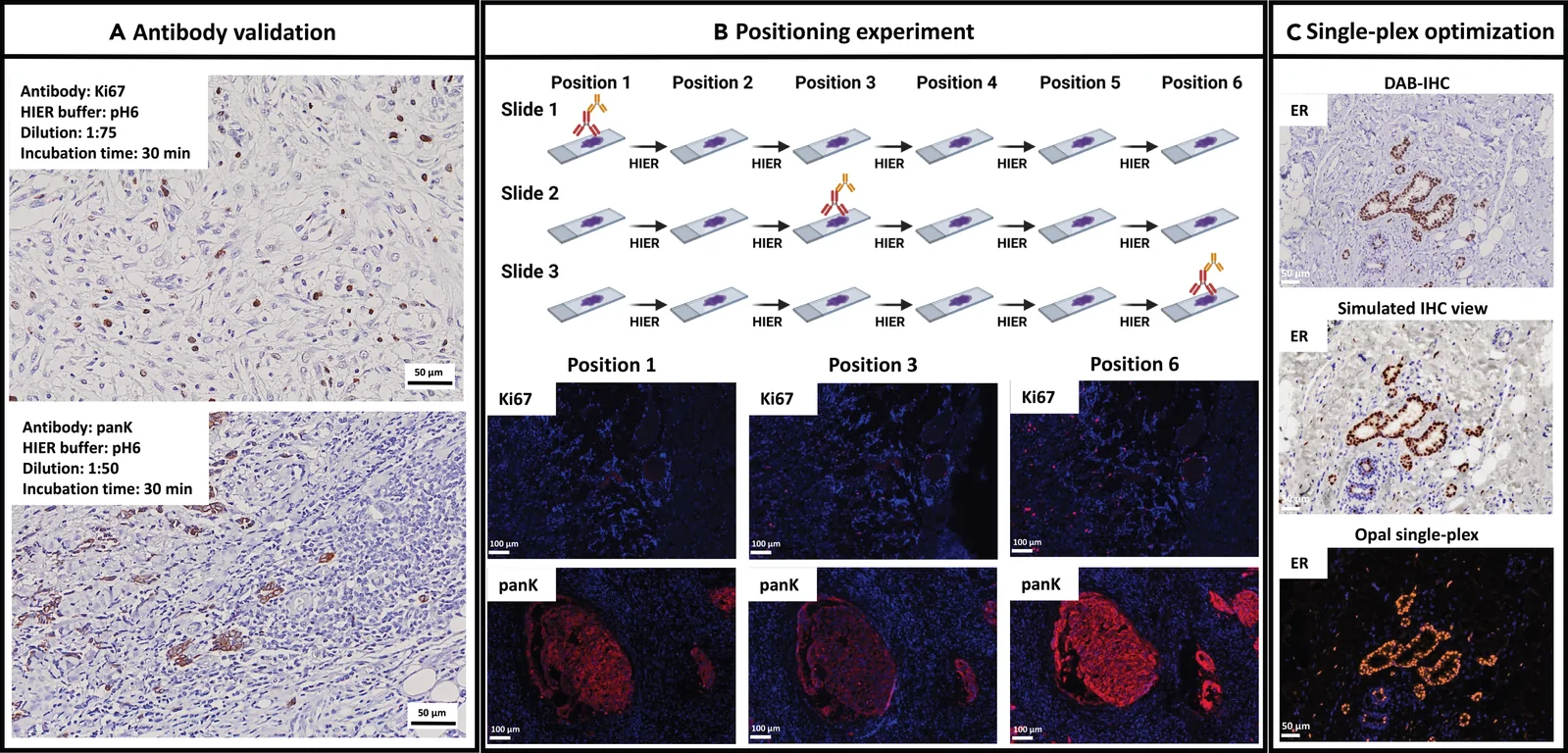
Panel establishment with antibody validation, positioning experiment and single-plex optimization (A) Antibody validation. Primary antibodies were validated using diaminobenzidine immunohistochemistry (DAB IHC) to determine optimal staining conditions. Validation included assessment of heat-induced epitope retrieval (HIER) buffer, antibody dilution, and incubation time. Representative DAB IHC images for Ki67 and pan-Keratin AE1/AE3 (panK) are shown with the corresponding optimized conditions. Scale bars indicate 50 µm. (B) Positioning experiment. Tissue sections were exposed to different numbers of HIER cycles to assess epitope sensitivity and determine the optimal order of antibodies within the multiplex panel. Representative examples for Ki67 and panK illustrate staining performance at different panel positions. Scale bars indicate 100 µm. (C) Single-plex optimization. Signal specificity for each antibody was verified using single-plex fluorescence staining. Consecutive serial sections were stained using DAB IHC and Opal single-plex fluorescence plus a simulated IHC view generated in QuPath using the ‘Invert background’ function. An example for the estrogen receptor (ER) antibody is shown. Scale bars indicate 50 µm.
  b. Prepare serial tissue sections for downstream optimization steps.
  c. Begin optimization using mild conditions (pH 6, recommended dilution, 30 min incubation) and adjust parameters as required.
    ***Note:*** Consultation with a pathologist is recommended to evaluate staining specificity and confirm appropriate staining patterns prior to multiplex implementation.
2. Positioning experiment.
  ***Note:*** A positioning experiment requires approximately 2 days. Ensure that all sections are processed under comparable conditions (Figure 2B).
  a. Test each antibody after multiple HIER cycles (e.g., after 1, 3, and 6 cycles) using serial sections to determine the optimal position within the multiplex staining sequence.
    ***Note:*** Antibody position within the multiplex staining sequence may affect staining quality because repeated HIER cycles can alter epitope accessibility or integrity.
  b. Use the same fluorophore for each antibody across all tested cycle positions.
    ***Note:*** Using the same fluorophore ensures that differences in signal intensity reflect antibody position effects rather than differences in fluorophore properties.
  c. Assign fluorophores independently to different antibodies according to panel design.
    ***Note:*** Fluorophore assignment between different antibodies does not affect the positioning analysis.
3. Single-plex optimization.
  a. Perform Opal single-plex staining using the validated antibodies (Figure 2C).
    ***Note:*** Compare staining pattern, fluorescence intensity, and SNR with chromogenic IHC staining as a reference.
  b. Assign Opal fluorophores according to marker abundance and spectral considerations.
    **CRITICAL:** Assign bright Opal fluorophores (e.g., Opal 480 and 520) to low-abundance markers to maximize signal detection despite increased tissue autofluorescence in these spectral channels. Assign dimmer fluorophores (e.g., Opal 690 and 780) to highly expressed markers to achieve balanced signal intensities across channels.
  c. Consider marker co-localization and spectral overlap during fluorophore assignment.
    **CRITICAL:** Appropriate fluorophore selection minimizes spectral bleed-through between channels.
  d. Optimize antibody dilution, Opal dilution, and incubation times to achieve optimal staining quality.
  e. Monitor fluorescence signal intensity and adjust exposure times to ensure balanced signal output across all channels.
4. Multiplex optimization.
  a. Combine all markers in a complete multiplex staining panel.
  b. Reassess fluorophore brightness, antibody positioning, and antibody dilutions in the context of the entire panel.
  c. Apply final adjustments to balance signal intensities and minimize spectral crosstalk across channels.
5. Plex-level extension.
  a. Perform iterative bleach-and-stain cycles to increase the plex level on the same tissue section.
  b. Reassess staining quality after each staining cycle.
  c. Adjust staining conditions as required before proceeding to the next cycle.

**CRITICAL:** Monitor tissue integrity throughout the procedure to ensure consistent staining quality after sequential bleach-and-stain cycles.

### Innovation

This protocol presents a fully integrated and robust sequential bleach-and-stain workflow that extends TSA-based Opal multiplex immunofluorescence beyond the standard 6-plex to higher plex levels. The iterative bleaching procedure is described in detail to ensure reproducibility, and the demonstrated 16-plex panel can be adapted to incorporate different or additional antibodies, enabling further extension beyond 16-plex.

While bleach-and-stain methods have already been described earlier such as IBEX^4^ and t-CyCIF,^3^ the novelty of this study lies in the integration of a complete workflow, encompassing low- to mid-plex staining, imaging and image processing within a single protocol paper.

A custom-developed Python script was implemented for image stitching, registration, and channel overlay, enabling sequential staining rounds to be aligned and merged into a unified high-plex dataset. The pipeline is specifically optimized for the Akoya image format and sequential whole-slide imaging across multiple staining rounds used in this study, enabling simplified cell-level and spatial analysis.

Together, this protocol provides a seamless end-to-end workflow from tissue sectioning to staining, imaging, and computational analysis, expanding multiplexing capacity on standard Akoya systems while leveraging open-source analysis tools and enabling flexible, high-content spatial data integration.

### Institutional permissions

The use of de-identified patient material for this study has been agreed by informed patient consent and has been approved by the ethics committee of the University of Tuebingen.

## Key resources table

**Table:** 

REAGENT or RESOURCE	SOURCE	IDENTIFIER
**Antibodies**		

Rabbit polyclonal anti-αSMA (1:100 dilution)	Abcam	Cat# ab5694, RRID:AB_2223021
Rabbit monoclonal anti-CD3 (1:100 dilution)	Cell Marque	Cat#103R-94
Mouse monoclonal anti-CD68 (1:300 dilution)	Cell Marque	Cat# 168M, RRID:AB_1158188
Rabbit polyclonal anti-phospho-Histone H2A.X (Ser139) (1:75 dilution)	Cell Signaling Technology	Cat# 2577, RRID:AB_2118010
Rabbit polyclonal anti-Cleaved Caspase-3 (1:75 dilution)	Cell Signaling Technology	Cat# 9661, RRID:AB_2341188
Rabbit monoclonal anti-CD4 (1:100 dilution)	Cell Marque	Cat# 104R, RRID:AB_1516770
Rabbit monoclonal anti-Granzyme B (1:50 dilution)	Cell Signaling Technology	Cat# 46890, RRID:AB_2799313
Rabbit monoclonal anti-CD8 (1:100 dilution)	Cell Marque	Cat# 108R, RRID:AB_2892088
Mouse monoclonal anti-pan-Keratin (AE1/AE3) (1:50 dilution)	Cell Signaling Technology	Cat# 67306, RRID:AB_3662123
Mouse monoclonal anti-Ki67 (1:100 dilution)	Agilent	Cat# M724029, RRID:AB_2687528
Rabbit monoclonal anti-Estrogen Receptor (ER) (ready-to-use)	Ventana Medical Systems	Cat# 790-4325; RRID:AB_2335977
Rabbit monoclonal anti-PD-L1 (1:100 dilution)	Cell Signaling Technology	Cat# 13684, RRID:AB_2687655
Monoclonal anti-CD20 (1:100 dilution)	Cell Marque	Cat# 120M-84, RRID:AB_1158145
Mouse monoclonal anti-CD31 (1:800 dilution)	Cell Signaling Technology	Cat# 3528, RRID:AB_2160882
Mouse monoclonal anti-FoxP3 (1:100 dilution)	eBioscience	Cat# 14-4777-82, RRID:AB_467556)
Rabbit monoclonal anti-CD45 (1:100 dilution)	Cell Signaling Technology	Cat# 13917, RRID:AB_2750898
Rabbit polyclonal anti-H3K27ac (1:400 dilution)	Abcam	Cat# ab4729, RRID:AB_2118291

**Biological samples**		

Human ovarian cancer FFPE blocks	Robert Bosch hospital	de-identified
Human lung cancer FFPE blocks	Robert Bosch hospital	de-identified
Human mantle cell lymphoma FFPE blocks	Robert Bosch hospital	de-identified
Human hepatocellular cancer FFPE blocks	Robert Bosch hospital	de-identified
Human pancreatic ductal adenocarcinoma	Robert Bosch hospital	de-identified
Human breast cancer FFPE blocks	Robert Bosch hospital	de-identified

**Chemicals, peptides, and recombinant proteins**		

Neo-Clear™ Xylene Substitute	Sigma-Aldrich	1.09843
Ethanol 99%	SAV Liquid Production	ETO-1000-99-1
Ethanol 96%	SAV Liquid Production	ETO-1000-96-1
Ethanol 70%	SAV Liquid Production	ETO-1000-70-1
Neutral buffered formalin (NBF) ROTI®Histofix 4%	Carl Roth	P087.4
TRIS Pufferan®	Carl Roth	4855.2
TRIS hydrochloride Pufferan®	Carl Roth	9090.3
Sodium chloride	Carl Roth	3957.1
Potassium chloride	Carl Roth	6781.3
TWEEN® 80	Sigma-Aldrich	8.22187
Hydrogen peroxide (H_2_O_2_) 30%	Carl Roth	CP26.5
Neo-Mount mounting medium	Sigma-Aldrich	1.09016
SlowFade™ Diamond Antifade Mountant	Invitrogen	S36963

**Critical commercial assays**		

Opal 6-Plex Manual Detection Kit	Akoya Biosciences	NEL811001KT

**Deposited data**		

Code for Stitching and Registration	This paper	https://github.com/Zha2Git/Stitching_Alignment_Tool
Image processing tutorial video	This paper	https://cloud.uni-jena.de/s/g92RdL4ejc2YGDM

**Software and algorithms**		

QuPath 0.4.4, 0.5.1, 0.6.0	Bankhead et al.^7^	https://qupath.github.io/
StarDist	Schmidt et al.^8^	https://github.com/stardist/stardist
inForm® Tissue Analysis Software 3.1.0	Akoya Biosciences	https://www.akoyabio.com/software-data-analysis/
Phenochart Whole Slide Viewer 2.2.0	Akoya Biosciences	https://www.akoyabio.com/support/software/
GraphPad Prism 11.0.0	GraphPad SOftware	https://www.graphpad.com/
Python 3.8.20	Python Software Foundation	https://www.python.org/
Anaconda Python	Python Software Foundation	https://docs.anaconda.com/anaconda/install/
Fusion 2.3.1	Akoya Biosciences	https://www.akoyabio.com/support/software/

**Other**		

Fluorescence microscope	Akoya Biosciences	PhenoImager Fusion
Full spectrum lamp	Waldmann	TND 1400/D50/D
Daylight lamp	Beurer	TL 30
3D Printer	Bambu Lab	X1-Carbon
Fully Automated Rotary Microtome	Leica Biosystems	RM2255
Microtome blades (80 × 8 mm)	Feather Safety Razor	A-35
Steam Pot	Rommelsbacher	DGS 855
EasyDip™ Slide staining jars	Simport Scientific	M900-12AS
EasyDip™ Slide staining rack	Simport Scientific	M905-12DGY
EnVision Detection Systems	Agilent	K500711-2
Transfer pipette 2 ml	Sarstedt	86.1176
Polysine Adhesion Slides	Epredia	J2800AMNZ
PAP Pen	Kisker Biotech	MKP-1
Microscope Cover Glasses (24 × 60 mm)	VWR	631-1575
4-well square tissue culture dish	Sarstedt	94.6077.307
Air blower	Giottos	GTAA1900

## Materials and equipment

**Table:** 10× TBS

Reagent	Final concentration [M]	Amount [g]
Sodium chloride	1.37	80
Potassium chloride	0.034	2
TRIS	0.028	3.39
TRIS hydrochloride	0.22	35
dH_2_O	N/A	Up to 1000 ml

Adjust the 10× TBS buffer to pH 7.4. Store at 4 °C for several months.

**Table:** 1× TBST

Reagent	Final concentration	Amount [ml]
10× TBS	1×	500
Tween 80	0.1%	5
dH_2_O	N/A	4500
Total	–	5000

Store the 1× TBST buffer at 21 °C and use within 2 weeks or at 4 °C for several months.

• Opal 6-Plex Manual Detection Kit includes 10x AR6 Buffer, 1× Antibody Diluent/Block, 1× Plus Amplification Diluent, 6 reactive fluorophores (Opal 480, Opal 520, Opal 570, Opal 620, Opal 690 and Opal 780), DMSO, 10× Spectral DAPI, 1× Opal Anti-Ms + Rb HRP.
• 1× Target Retrieval Solution (Citrate, pH 6): dilute 10x Target Retrieval Solution (Citrate, pH 6) at 1:10 with dH_2_O. Store at 4 °C and use within 3 months.
• 1× Target Retrieval Solution (pH 9): dilute 10× Target Retrieval Solution (pH 9) at 1:10 with dH_2_O. Store at 4 °C and use within 3 months.
• Opal fluorophores (Opal 480, Opal 520, Opal 570, Opal 620 and Opal 690): reconstitute with 75 μl Dimethyl sulfoxide (DMSO). Store at 4 °C in dark for up to 3 months.
• Opal fluorophore (Opal 780): reconstitute with 300 μl dH_2_O. Store at 4 °C in dark for up to 3 months.
• Opal fluorophore working solution: dilute reconstituted Opal fluorophores at 1:100 in 1× Plus Amplification Diluent, except Opal 780, which is diluted at 1:25 with Ab diluent/blocking. Prepare working solution for volume needed.
• DAPI working solution: dilute 3 drops DAPI in 1 ml of 1× TBS. Store at 4 °C in dark for up to 2 weeks.

Fluorophore and Autofluorescence (AF) bleaching solutions: Prepare bleaching solution shortly before use. The final volume of solution is 10 ml with the following working concentrations:

• AF bleaching solution (4.5% H_2_O_2_): 8.5 ml 1× TBS, 1.5 ml of 30% (wt/vol) H_2_O_2_.
• Fluorophore bleaching solution (3% H_2_O_2_): 9 ml 1× TBS, 1 ml of 30% (wt/vol) H_2_O_2_.

## Step-by-step method details

Unless otherwise specified, all incubations were performed at 21 °C. Reagent volumes were adjusted to fully cover the tissue section (typically 100–300 μl per slide).

The washing steps were performed by placing the slides vertically in a slide processing jar filled with sufficient solution to fully cover the tissue section.

### Sectioning and preparing FFPE tissue slides

**Timing: 2 h to overnight**

This section describes how to cut and prepare FFPE tissue sections for the next step.

***Note:*** Prior to sectioning, store blocks at −20 °C for at least 30 min to allow cooling, which facilitates smooth cutting and minimizes wrinkling or curling.

1. Cut 3 μm FFPE sections with a microtome.

***Note:*** The thickness of the sections is variable; a thickness between 2–5 μm usually has no noticeable effect on the staining.

2. Transfer the sections to a water bath preheated to 35–40°C to allow them to stretch, then mount them onto microscope slides.

***Note:*** Use electrostatically and biochemically adhesive slides to improve tissue adherence.

**CRITICAL:** For samples rich in fat or connective tissue, e.g. breast tissue, reduce the water bath temperature to 30°C and transfer sections promptly onto the slide to maintain structural integrity and minimize tissue diffusion.

3. Dry the slides at room temperature for 10 min and afterwards incubate slides at 56–60°C for 8–24 h.

***Note:*** Prolonged incubation enhances tissue adherence; however, incubation should not exceed 24 h as longer incubation may lead to heat-induced epitope modification or masking and reduced tissue quality.

**Pause point:** Freshly cut tissue sections can be used immediately and are always recommended. For storage, keep the slides at room temperature for 1 to 2 weeks in a dry, dust-free slide box; for up to 1 to 2 months in a desiccator, or at −20 °C or −80 °C for several months. Compared with freshly cut FFPE sections, long-term storage at −20 °C generally results in comparable morphology but may produce modest reductions in antigenicity. We examined tissue samples from ovarian, lung, and breast cancer that had been stored for up to one year without any visible deterioration in signal quality.

### Rehydration and antigen retrieval

**Timing: 2–3 h**

This section describes the rehydration of tissue sections and antigen retrieval process performed with a steam pot.

4. Put slides in the slide staining rack.

***Note:*** Allow slides stored at −20 °C to equilibrate to room temperature before proceeding.

5. Dewax slides in a slide processing jar filled with about 150 ml Neo-Clear Xylene Substitute solution at room temperature for 20 min.
6. Rehydrate slides in descending alcohol series.
  a. 100% ethanol for 3 min.
  b. 96% ethanol for 3 min.
  c. 70% ethanol for 3 min.
7. Rinse slides in dH_2_O.
8. Fix slides in 4% neutral buffered formalin for 20 min.
  a. While fixation, prepare 1× Target Retrieval Solution (pH 6 or pH 9) in a slide processing jar and prewarm in a steam pot for 20 min.

***Note:*** We typically use pH 9 for the first round to achieve effective epitope retrieval and pH 6 for subsequent rounds of antibody stripping. Depending on the antibodies used, pH 9 may not be necessary and should be avoided, as it affects tissue integrity more than pH 6. Several pH 6 rounds can also replace the pH 9 treatment (Troubleshooting Problem 1).

9. Rinse slides in dH_2_O.
10. Antigen Retrieval:
  a. Transfer slides into a preheated slide processing jar filled with 1x Target Retrieval Solution (pH 6 or pH 9) in a steam pot.
  b. Loosely cover the jar with lid and cook the slides at 95 °C in the buffer for 30 min.

**CRITICAL:** Prewarming is essential for sufficient HIER step.

***Alternatives:*** HIER can also be performed using a water bath, pressure cooker, or microwave.

11. Take the jar out of the steam pot, open the lid and cool down at room temperature for 20 min.

***Note:*** Do not let slides dry out.

12. Wash 2 to 3 times in 1× TBST for 3 min each.

**Pause point:** Slides can be stored at 4°C in TBST for up to 2 days.

### Autofluorescence reduction

**Timing: 1.5 h**

This section describes optional steps to reduce autofluorescence (AF) before starting the staining process.

**Figure 3:**
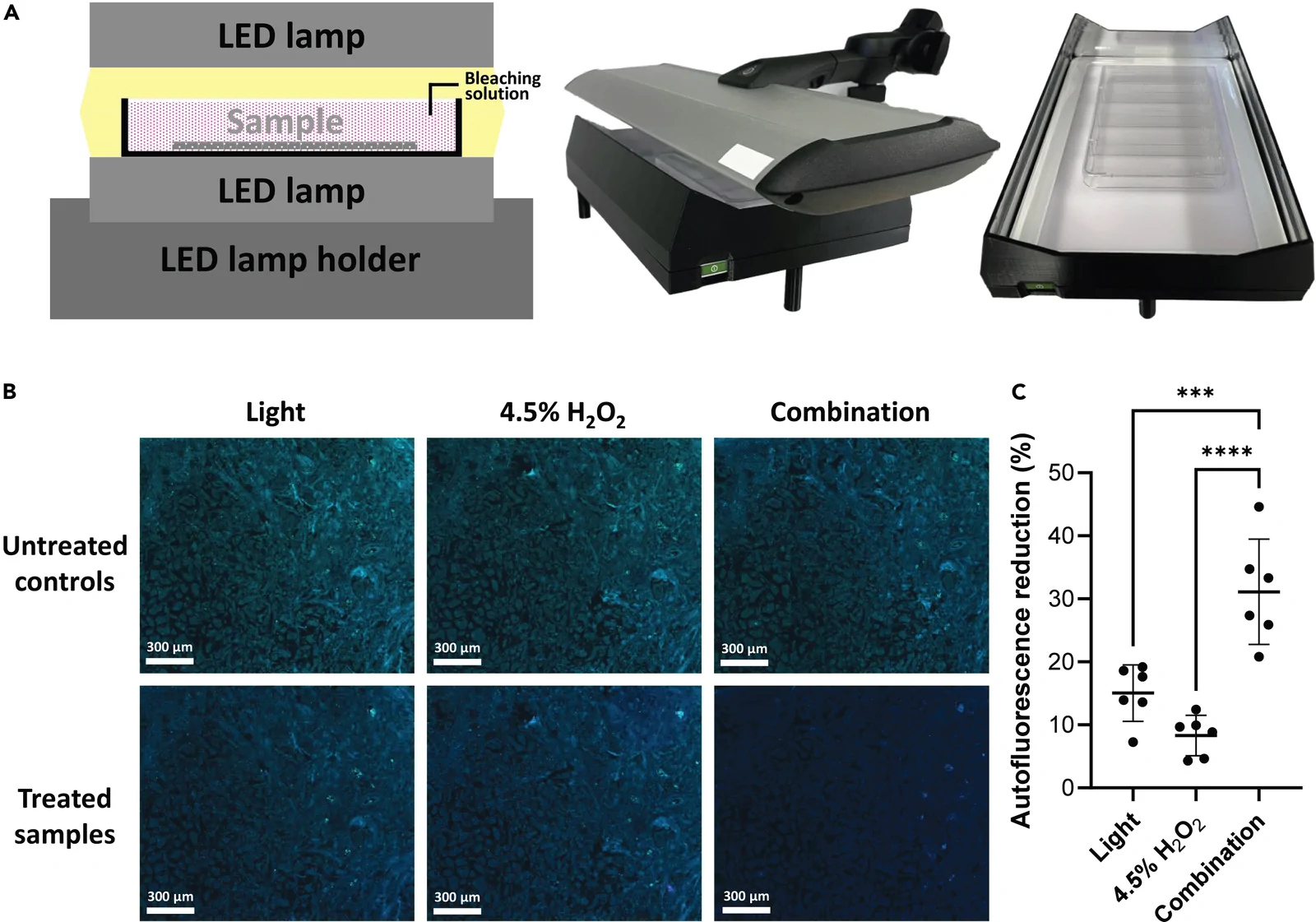
Autofluorescence reduction by photobleaching in hydrogen peroxide solution (A) Setup used for autofluorescence reduction and fluorophore inactivation. Tissue slides are placed in a 4-well square tissue culture dish containing hydrogen peroxide (H_2_O_2_) solution and illuminated from above and below using LED lamps. (B) Representative overlay images of all fluorescence channels acquired from formalin-fixed, paraffin-embedded breast cancer tissue sections subjected to different autofluorescence reduction treatments (light exposure, 4.5% H_2_O_2_, or a combination of both) compared to corresponding untreated control sections. Scale bars indicate 300 µm. (C) Quantification of autofluorescence intensity reduction across treatment conditions. Median autofluorescence intensity was measured across all acquired fluorescence channels in defined regions of interest in serial tumor sections using QuPath, as the median provides a robust measure of the typical autofluorescence signal and is less affected by isolated bright structures or imaging artifacts. Autofluorescence reduction was calculated as percentage decrease relative to untreated controls. Statistical analysis was performed using one-way ANOVA with Šídák’s multiple comparisons test. Data are presented as mean ± SD with individual ROI values shown. Asterisks indicate statistical significance: ∗∗∗*p* ≤ 0.001 and ∗∗∗∗*p* ≤ 0.0001

***Note:*** For this purpose, the samples are placed in a bleaching solution between two LED lamps (Figure 3A). The lamp holder was designed in-house and can be printed with a 3D printer. The STL file for 3D printing can be downloaded here. The pre-bleaching step is recommended for tissue with high AF, including those abundant in fibers or blood vessels (Figure 3), and can be performed in addition to AF correction based on an external slide (Step 56). Quantitative analysis of serial tissue sections demonstrated that the combined application of H_2_O_2_ and light exposure resulted in a significantly greater reduction of autofluorescence than either treatment alone (Figures 3B and 3C), supporting the use of the combined chemical photobleaching approach described in this protocol.

**CRITICAL:** Carefully consider this step as it may affect the integrity of the tissue.

**CRITICAL:** Perform AF reduction after the first cooking step (Step 10–12). Bleaching followed by additional heating may cause increased tissue detachment.

**CRITICAL:** Hydrogen peroxide should be handled with appropriate personal protective equipment, including gloves and eye protection. Light exposure systems should be used according to manufacturer safety guidelines.

***Note:*** Depending on the tissue type, different parameters like the composition of the bleaching solution or the exposure time can be adjusted. The standard protocol for ovarian, lung and breast cancer tissue is described below.

13. Wash 3 times in 1× TBS for 3 min each.
  a. While washing, prepare 8 ml of AF bleaching solution (4.5% H_2_O_2_ in 1× TBS) per slide.

**CRITICAL:** Prepare bleaching solution shortly before use.

14. Autofluorescence Reduction (50 min).
  a. Place slides flat in the 4-well square tissue culture dish and completely cover the tissue with 8 ml AF bleaching solution per slide.
  b. Position the container between the lamps to maximize light exposure.
    i. Place the closed 4-well square tissue culture dish directly on the daylight lamp.
    ii. Arrange the full spectrum lamp above the lid of the plate (Figure 3A).
  c. Incubate in AF bleaching solution with exposure to light for 45 min.
15. Wash 1 time in 1× TBS for 3 min.
16. Wash 2 to 3 times in 1× TBST for 3 min.

**Pause point:** Slides can be stored at 4 °C in TBST for up to 2 days.

### Tissue staining with Opals 480–690

**Timing: 2.5–3 h per Opal fluorophore**

This section describes tissue staining for the Opal fluorophores 480–690.

17. Blocking:
  a. Place slides in a humidified chamber and drop 100–300 μL (2 to 3 drops) blocking buffer (1× Antibody Diluent/Block) onto the slides until the tissue is fully covered.
  b. Incubate the slides at room temperature for 10 min.
  c. While blocking, prepare 100–300 μL of primary antibody working solution (primary antibody diluted in 1× Antibody Diluent/Block).

***Note:*** Ensure that the tissue section is fully covered with solution without air bubbles.

18. Wash 1 time in 1× TBST for 3 min.
19. Primary Antibody Incubation:
  a. Place slides in a humidified chamber and pipette 100–300 μL of primary antibody working solution onto the slides.
  b. Incubate the slides at room temperature for 30 min.

***Note:*** Incubate based on previously optimized chromogenic DAB IHC and single-plex staining results. The standard incubation time is 30 min.

20. Wash 2 to 3 times in 1× TBST for 3 min each.
21. Secondary Antibody Incubation:
  a. Place slides in a humidified chamber and drop 100–300 μL (2 to 3 drops) secondary antibody (1× Opal Anti-Ms + Rb HRP) onto the slides.
  b. Incubate the slides at room temperature for 10 min.
22. Wash 2 to 3 times in 1× TBST for 3 min each.
  a. While washing, prepare 1× Target Retrieval Solution (Citrate, pH 6) in slide processing jar and prewarm in steam pot for 20 min for antibody stripping if next staining round is performed.
  b. While washing, prepare 100–300 μL Opal fluorophore working solution (Opal fluorophore 480, 520, 570, 620 or 690 at 1:100 in 1× Plus Amplification diluent).
23. Signal Amplification:
  a. Place slides in a humidified chamber and pipette 100–300 μL of Opal fluorophore working solution onto the slides.
  b. Incubate the slides at room temperature for 10 min.

***Note:*** Use Opal fluorophore according to the specified sequence determined by the results of the positioning test and the Opal single-plex optimization.

**CRITICAL:** Use Opal 780 as the last fluorophore, as its mechanism of action is slightly different.

24. Wash 2 to 3 times in 1× TBST for 3 min each.

**Pause point:** Slides can be stored at 4 °C in TBST for up to 2 days.

25. Antibody Stripping:
  a. Place slides into a preheated slide processing jar filled with 1x Target Retrieval Solution (Citrate, pH 6).
  b. Loosely cover the jar with lid and cook the slides at 95°C in the buffer for 30 min.

**CRITICAL:** Prewarm is essential for sufficient HIER step.

***Alternatives:*** HIER can also be performed using a water bath, pressure cooker, or microwave.

26. Take the jar out of the steam pot and cool down at room temperature for 20 min.

***Note:*** Do not let slides dry out.

27. Wash 2 to 3 × in 1× TBST for 3 min each.

**Pause point:** Slides can be stored at 4 °C in 1× TBST for up to 2 days.

Repeat the steps tissue staining with Opals 480–690 **(steps 17–27)** for the remaining markers paired with Opal fluorophores 480-690 using the previously determined Antibody-Opal pairings. For the last fluorophore Opal Polaris 780 continue with Tissue staining with Opal 780.

### Tissue staining with Opal 780

**Timing: 3.5–4 h**

This section describes tissue staining with the last fluorophore Opal Polaris 780.

Repeat the **steps 17–21 (Blocking - Primary + Secondary Antibody incubation**).

28. Wash 2 to 3 times in 1× TBST for 3 min each.
  a. While washing, prepare 100–300 μL Opal TSA-DIG working solution (TSA-DIG at 1:100 in 1× Plus Amplification Diluent).
29. Signal Amplification of Opal TSA-DIG:
  a. Place slides in a humidified chamber and pipette 100-300 μl of Opal TSA-DIG working solution onto each slide.
  b. Incubate the slides at room temperature for 10 min.
  c. While incubating, prepare 1× Target Retrieval Solution (Citrate, pH 6) for antibody stripping and prewarm in steam pot for 20 min.
30. Wash 2 to 3 x in 1× TBST for 3 min each.
31. Antibody Stripping:
  a. Place slides into a preheated slide processing jar filled with 1× Target Retrieval Solution (Citrate, pH 6).
  b. Loosely cover the jar with lid and cook the slides at 95°C in the buffer for 30 min.

**CRITICAL:** Prewarming is essential for sufficient HIER step.

***Alternatives:*** HIER can also be performed using a water bath, pressure cooker, or microwave.

32. Take the slide processing jar out of the steam pot, open the lid and cool down at room temperature for 20 min.

***Note:*** Do not let slides dry out.

33. Wash 2 to 3 times in 1× TBST for 3 min each.
  a. While washing, prepare Opal fluorophore 780 working solution at **1:25** in 1× Antibody Diluent/Block.
34. Opal 780 Signal Generation:
  a. Place slides in a humidified chamber and pipette 100-300 μl Opal Polaris 780 working solution onto each slide.
  b. Incubate the slides at room temperature for **1 h.**

***Note:*** Do not perform HIER after this step.

### Counterstain and coverslip

**Timing: 30 min**

This section describes counterstaining with DAPI and coverslipping slides with mounting medium.

35. Wash 2 to 3 times in 1× TBST for 3 min each.
  a. While washing, dilute 3 drops DAPI in 1 mL of 1× TBS.
36. Counterstain:
  a. Place slides in a humidified chamber and add 150 μL DAPI working solution onto each slide.
  b. Incubate the slides at room temperature for 5 min.

***Note:*** Discard any remaining DAPI working solution.

37. Wash 1 time in 1× TBST for 2 min.
38. Wash 1 time in dH_2_O for 2 min.
39. Air dry the slides, protected from light and dust.
40. **Coverslip**: Drop 2 to 3 drops SlowFade™ Diamond Mounting Medium onto each slide and coverslip slides.

***Note:*** Be careful to avoid introducing bubbles. Allow the mounting medium to spread evenly beneath the coverslip, covering the entire tissue.

***Alternatives:*** For a cost-effective liquid mounting option, slides can be mounted with 10% (v/v) glycerol in 1× TBS.^3^^,^^9^ Apply approximately 200 μl to the tissue before coverslipping.

**CRITICAL:** Use liquid mounting medium to allow easy removal of cover glass for subsequent staining rounds.

***Note:*** Use air blower to prevent dust before mounting the slides.

### Imaging

**Timing: 1 h**

This section describes imaging of the stained slides using Akoya PhenoImager platform.

***Note:*** The system is equipped with an LED light source, supports both 20× and 40× objectives and uses a high-speed camera detector for multispectral image acquisition across a wavelength range from 440–780 nm.

41. Turn on the computer and switch on the PhenoImager Fusion microscope.
42. Launch the Fusion 2.3.1 software.
43. Set up PhenoImager slide carriers.
  a. Insert each slide carefully into the desired position until it rests against the spring-loaded tab.
  b. Load up to four slides per slide carrier.
  c. Insert the slide carrier into the automated XYZ stage, pushing it until it fully passes through the gate.
  d. Optional: Reference settings measurements.
    i. Open ‘Fusion Dashboard’ from the software homepage.
    ii. Brightfield References: Insert the reference carrier to acquire new brightfield references; perform biweekly (∼5 min).
    iii. Fluorescence References for Coverslipped Slides: Insert the reference carrier to acquire new fluorescence references; perform monthly (∼20 min).
44. Create or Load Fluorescence Protocols.
  a. Click ‘Brightfield and Fluorescence Protocols’.
  b. Create new Protocol: Click ‘New’ and the Create Protocol window opens.
    i. Enter the Protocol name.
    ii. Select Imaging Mode ‘Fluorescence’.
    iii. Select Filter Set ‘Opal 7-color’.
    iv. Select an existing study or create a new study.
    v. Click ‘Create Protocol’.
  c. Alternative: Load an existing Protocol.
    i. Click ‘Load’ and select a previously created study.
45. Edit Fluorescence Protocol.
  a. Adjust the Pixel Resolution to 20× or 40×.
  b. Click ‘Select Bands’.
    i. Choose the filters for scanning. To remove a band, deselect its channel.
    ii. Adjust the colors associated with each band; Phenochart displays the scan using these colors.
    iii. Click ‘OK’ to confirm.
  c. Set Exposure Times.
    i. Click ‘Scan Exposures’ to open the window.
    ii. Select the slide from the drop-down list.
    iii. Navigate the XY Stage to the sample position and fine-tune the position using the arrow buttons (small movements) or sliders (larger movements).
    iv. Adjust the Z stage slider to bring the live view approximately into focus and press ‘Autofocus’ to confirm.
    v. View the first filter live.
    vi. Click ‘Autoexpose’ to let the system determine optimal exposure.
      ***Optional:*** Override the exposure by entering a value (0.1–2000 ms) in the highlighted text box.
    vii. Repeat for all filter bands, adjusting slide position as needed to optimize signal.
  d. Save the Protocol.
    i. Click ‘Back’ to return to ‘Edit Protocol’ window.
    ii. Save protocol.
46. Configure Slide Scanning.
  a. Return to the PhenoImager Fusion main menu.
  b. Click ‘Scan Slides’ to open the ‘Editing Slides’ window.
  c. For each slide, enter the following.
    i. Task: Choose ‘Scan Slide’ or select ‘Ignore’ if no slide is present.
    ii. Study: Select a study.
    iii. Protocol: Select the corresponding saved protocol.
    iv. Slide ID: Enter the slide identifier.
47. Start Scanning.
  a. Ensure at least one slide is ready and click ‘Scan Slides’ to begin scanning.
  b. Monitor the scan progress for each slide.
  c. When scanning completes, review the pop-up window displaying scan results.
48. Remove Slides.
  a. Remove the PhenoImager slide carrier from the stage.
  b. Remove the slides from the carriers.
49. Switch off the PhenoImager Fusion.

### Inactivation of fluorophores

**Timing: 3 h**

**Figure 4:**
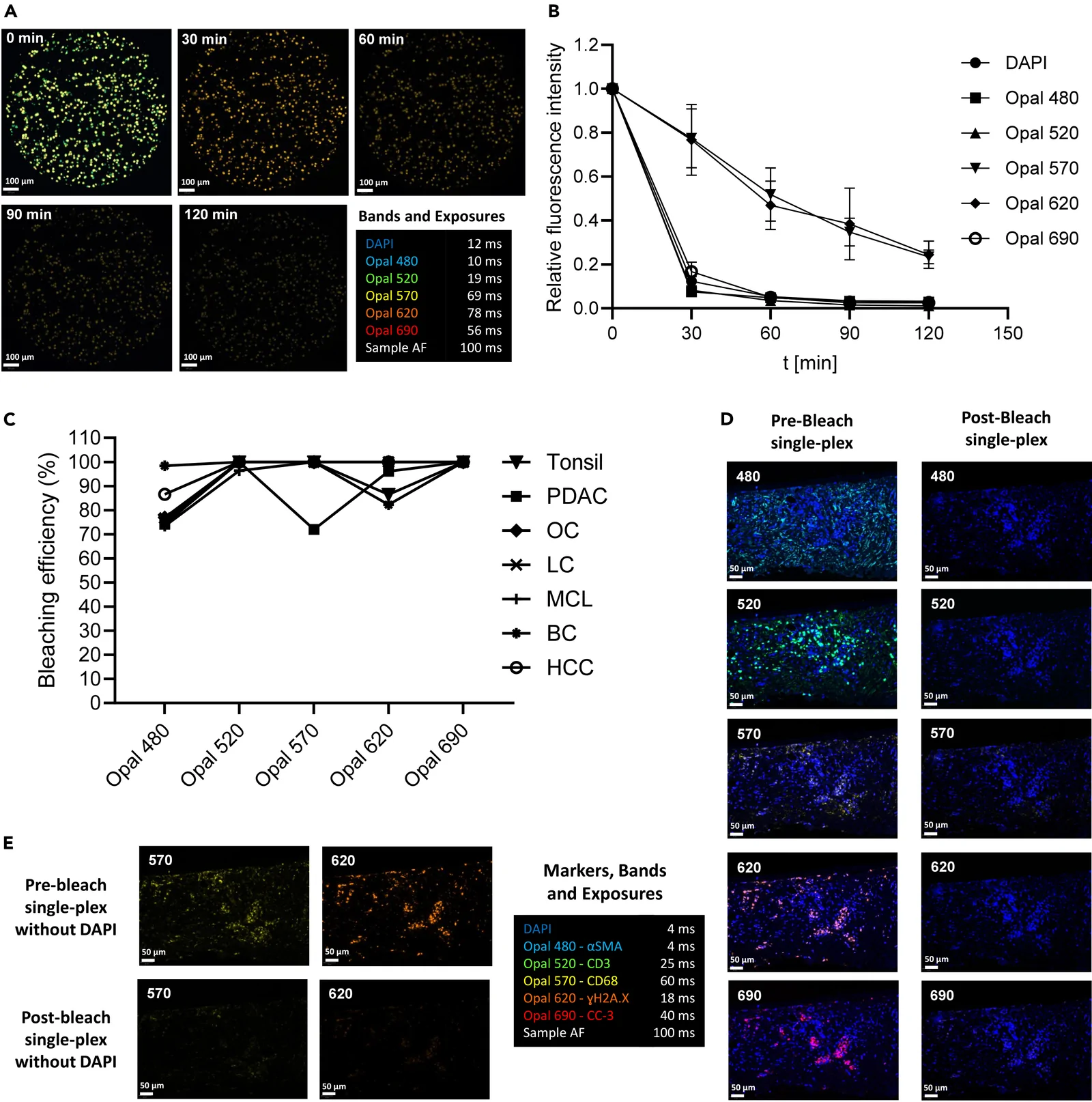
Fluorophore inactivation by photobleaching in hydrogen peroxide solution (A) After completion of the first staining round, fluorophores on the FFPE tissue sections are inactivated using combined light exposure and 3% H_2_O_2_. Fluorophore inactivation is illustrated using an FFPE embedded cell pellet of HCC70 breast cancer cell line. Images were acquired using fixed auto-exposure settings defined at the start of the inactivation procedure (0 min). Representative images are shown at 0, 30, 60, 90 and 120 min of photobleaching for Opal fluorophores 480-690 and DAPI. Scale bars indicate 100 µm. (B) Quantification of fluorophore inactivation over time, displayed as the reciprocal of the normalized auto-exposure time at t = 0 min (relative fluorescence intensity), which provides a relative measure of remaining signal intensity. Measurements were obtained for DAPI and Opal fluorophores 480-690 throughout the bleaching period time points (0, 30, 60, 90 and 120 min). Auto-exposure mode was used to maintain measurements within the linear dynamic range and allow comparison across fluorophores. Data are presented as mean ± SD (n = 2). (C) Quantification of Opal fluorophore signal reduction after chemical photobleaching across different tissue types, including tonsil, pancreatic ductal adenocarcinoma (PDAC), ovarian cancer (OC), lung cancer (LC), mantle cell lyphoma (MCL), breast cancer (BC) and hepatocellular carcinoma (HCC). Mean fluorescence intensity was quantified for each Opal channel in defined regions of interest across all tissue entities using QuPath to assess the overall residual fluorophore signal after chemical photobleaching. For each Opal channel (480-690), signal reduction was calculated as the percentage decrease in fluorescence intensity before versus after bleaching, where higher values indicate more effective fluorophore inactivation. (D) Representative ovarian cancer images comparing pre-bleach 6-plex staining with post-bleach single-plex images for Opal fluorophores 480–690. For visualization purposes, DAPI intensity in post-bleach images was adjusted to facilitate comparison with the corresponding pre-bleach images. Scale bars indicate 50 µm. For detailed information on antibody-fluorophore assignments, see Table 1. (E) Magnified views of the Opal 570 and Opal 620 channels shown without DAPI overlay. Despite comparatively lower bleaching efficiency of these fluorophores, no appreciable residual tissue signal remained after bleaching, indicating sufficient fluorophore inactivation for subsequent staining cycles. Scale bars indicate 50 µm.

This section describes the inactivation of fluorophores after each staining cycle to enable subsequent bleach-and-stain cycles (Figure 4).

***Note:*** Fluorophore inactivation is achieved by combined light exposure and 3% H_2_O_2_, resulting in efficient signal removal while preserving tissue morphology for subsequent staining cycles. Figure 4 demonstrates the kinetics of fluorophore inactivation over time, quantitative bleaching efficiency across multiple Opal fluorophores and diverse FFPE tissue types, as well as representative pre- and post-bleaching images confirming effective signal removal prior to the next staining cycle.

50. Remove the coverslips.
  a. Put the slides vertically in 1× TBS in a slide processing jar without rack for 5 min.
  b. Gently pull the slides vertically out of the TBS solution and leave the coverslip in the buffer.

**CRITICAL:** If the coverslip does not release by itself when pulling it out, leave the slide in the wash buffer for a longer period. Avoid removing the coverslip manually, since manual removal can damage the tissue.

**CRITICAL:** Hydrogen peroxide should be handled with appropriate personal protective equipment, including gloves and eye protection. Light exposure systems should be used according to manufacturer safety guidelines.

51. Wash 3 times in 1× TBS for 3 min each.
  a. While washing, prepare 8 ml of fluorophore bleaching solution (3% H_2_O_2_ in 1× TBS) per slide.

**CRITICAL:** Prepare bleaching solution shortly before use.

52. First Round: Inactivation of Opal fluorophores (65 min).
  a. Place slides flat in the 4-well square tissue culture dish and completely cover the tissue with 8 mL of fluorophore bleaching solution per slide.
  b. Position the container between the lamps to maximize light exposure.
    i. Place the closed 4-well square tissue culture dish directly on the daylight lamp.
    ii. Arrange the full spectrum lamp above the lid of the plate (Figure 3A).
  c. Incubate in fluorophore bleaching solution with exposure to light for 60 min.

***Note:*** Prepare fluorophore bleaching solution for the second round of bleaching shortly before use.

53. Second Round: Inactivation of Opal fluorophores (65 min).
  a. Replace the fluorophore bleaching solution and incubate again under light exposure for 60 min.
54. Wash 1 time in 1× TBS for 3 min.
55. Wash 3 times in 1× TBST for 3 min each.

***Note:*** Bubbles form during the bleaching process due to an oxidation process.

**CRITICAL:** Hydrophobic barrier pens can interfere with subsequent staining cycles.^6^^,^^9^ When using these pens, completely remove any residues from the slide before bleaching the fluorophores and performing the subsequent cooking steps.

### Further rounds of tissue staining

**Timing: 2.5–3 h per Opal fluorophore**

For each subsequent staining round, repeat the steps described in tissue staining with Opals 480–690 with a final counterstain and coverslip and imaging.

***Note:*** Opal 780 cannot be used for further rounds because the TSA-DIG remains bound and the corresponding marker is therefore re-stained during incubation with Opal 780.

### Unmixing with inForm Tissue Analysis Software

**Timing: 15 min**

This section describes the unmixing process, which removes autofluorescence and separates overlapping fluorophore spectra to achieve high signal-to-noise ratios.^10^ A step-by-step tutorial video demonstrating image unmixing is available at https://cloud.uni-jena.de/s/g92RdL4ejc2YGDM.

***Note:*** This video demonstrates the image unmixing workflow using inForm® Tissue Analysis Software. 1) Generation of the unmixing library - Phenochart ROI selection. 2) Generation of the unmixing library - inForm. 3) Unmixing of stained sections - Phenochart ROI selection. 4) Unmixing of stained sections - inForm.

***Note:*** The inForm® Tissue Analysis Software version 3.1.0 is used to unmix the images.

56. Prepare an unstained section from the same tumor type to generate the unmixing library.

***Note:*** Perform antigen retrievals, blocking, primary and secondary antibody incubations in all cycles, with TBST washes in between. Optionally, include monoplex-stained controls to refine spectral accuracy. A detailed description of the preparation of unstained slides for the unmixing process can be found in the SOP for AF slides.

57. Generate unmixing library with an unstained section.
  a. Open the unstained image in Phenochart.
  b. Log in with a username.
    ***Note:*** Entering initials is sufficient, as this step only serves to track modifications.
  c. Navigate to Stamp > inForm Projects > Set stamps.
  d. Close the window (settings are saved automatically).
  e. Open inForm® Tissue Analysis Software.
    i. Go to File > Open > Image and select the unstained section.
    ii. Click ‘Edit Markers and Colors’ to enter marker names and assign colors.
    iii. Use the ‘pen tool’ to ‘select autofluorescence from the image and add to the library’ by drawing a line through representative autofluorescent regions on the image.
    iv. Click ‘Prepare image’.
    v. Use the ‘eye icon’ to inspect results.
    vi. Save via File > Save > Algorithm.
      ***Note:*** The saved unmixing algorithm is required for subsequent stained sections from the same tumor type.
58. Apply unmixing to stained sections.
  a. Open the stained image in Phenochart.
  b. Log in with a username.
  c. Select ROI > Acquisition > inForm Batch and draw a line around the tissue.
  d. Open inForm® Tissue Analysis Software.
    i. Select ‘Batch Analysis’.
    ii. Under ‘Batch Algorithm or Project’, select ‘Browse’ and load the algorithm created with the unstained section.
    iii. Under ‘Export Directory’, select ‘Browse’ and choose the folder for saving results files.
    iv. Under ‘Images to Export’, tick ‘Component Images’ to export data as multi-image TIFFs.
    v. Under Input Files, select ‘Add slides acquired by Fusion for inForm batch processing’ to select images to be analyzed.
    vi. Click Run.
  e. Results of unmixed sections.
    i. Component image tiles, which must be stitched to generate the full image.

***Note:*** If using the PhenoImager HT 2.0, manual unmixing is not required because unmixing is integrated during scanning.

### Stitching and registration

**Timing: 60 min per slide (raw data 5 GB.tif images)**

**Figure 5:**
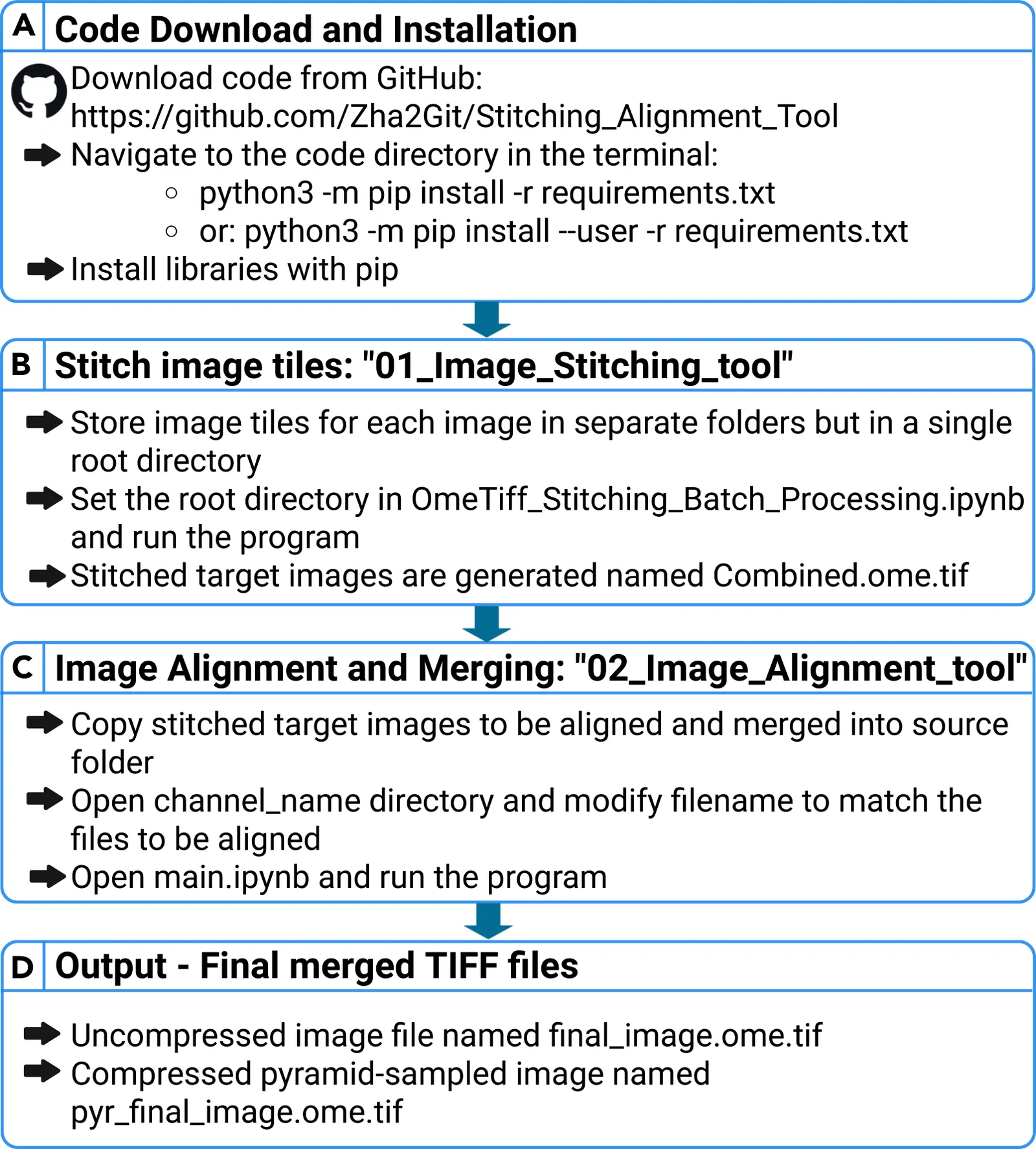
Workflow for image stitching, alignment and merging (A) The stitching and alignment tools are downloaded from GitHub and installed in a local Python environment using pip. (B) Image tiles are organized into subfolders within a single root directory and batch-stitched using “01_Image_Stitching_tool”, generating stitched OME-TIFF files. (C) Image alignment and merging using “02_Image_Alignment_tool”. Stitched images are copied to the source directory, channel names are assigned, and the alignment script “main.ipynb” is executed to align channels and merge image layers. (D) The pipeline outputs a final merged uncompressed OME-TIFF and a compressed pyramid-sampled image.

This section describes how images from consecutive staining cycles are stitched, aligned and merged into a single multi-layer image for downstream analysis (Figure 5). A step-by-step tutorial video demonstrating image stitching, alignment and overlay is available at https://cloud.uni-jena.de/s/g92RdL4ejc2YGDM.

***Note:*** This video demonstrates the computational workflow for image stitching, registration, and overlay using the Python/Jupyter Notebook pipeline described in this protocol. 1) Image stitching. 2) Image alignment and overlay.

***Note:*** The complete workflow is implemented in the provided Python & Jupyter Notebook scripts. Recommended computer hardware is at least 32 GB RAM.

**CRITICAL:** Install a functional Python environment before starting the workflow.

***Note:*** We recommend installing the Anaconda Distribution following the instructions at Installing Anaconda Distribution, which includes Python and Jupyter Notebook and provides detailed installation guides, video tutorials, and FAQs for different operating systems (Windows, macOS, and Linux). The workflow can also be executed in an integrated development environment (IDE), such as Visual Studio Code on Windows.

**CRITICAL:** To ensure compatibility with the provided scripts, use Python 3.8.20 together with the following package versions, which were used to develop and validate the workflow: numpy 1.22.2, tifffile 2022.2.9, scikit-image 0.19.2, pystackreg 0.2.5, pandas 1.4.1, Jupyter Notebook and ipykernel. Other software versions may require minor code adaptations.

59. Code Download and Installation.
  a. Download the code from the GitHub repository Stitching_Alignment_Tool.
    ***Note:*** The GitHub repository includes a user guide README.md file that provides detailed, step-by-step instructions.
  b. Install the code within the local Python environment.
  c. Open a terminal and navigate to the code directory using >cd command. This directory serves as the working directory.
  d. Determine whether administrator (root) privileges are available.
    i. If yes, install the required libraries by running the following command from the code directory:
      >bash
      >python3 -m pip install -r requirements.txt
    ii. If not (e.g., on a shared server or multi-user system), run:
      >bash
      >python3 -m pip install --user -r requirements.txt
      ***Note:*** This command installs all required Python libraries using the default Python package manager (pip).
60. Stitch Image Tiles using “01_Image_Stitching_tool“.
  a. Verify that image tiles generated after spectral unmixing of stained sections contain the coordinates of the lower-right vertex in their filenames (e.g., “_[x_coordinate, y_coordinate]_“).
    i. For multiple images to be stitched, store the tiles of each microscope image in a separate folder.
    ii. Store all separate folders within a single root directory.
  b. Set the root directory in “OmeTiff_Stitching_Batch_Processing.ipynb”.
  c. Run the program.

***Note:*** The program performs batch stitching for all subfolders within the root directory and records the processing status in a log file.

***Note:*** For each task, the program iterates through all TIFF files, removes files containing keywords, and filters unstitched tile files.

***Note:*** The size of the stitched target image is calculated based on the coordinates of all tile files, determining the correct position of each tile in the final image.

***Note:*** The program generates stitched target images while preserving layer names from the tile files. Output files are saved in the corresponding subfolders and named “XXXX_Combined.ome.tif“. These stitched images are provided for the subsequent image alignment and layer merging operations.

61. Image Alignment and Merging with “02_Image_Alignment_tool“.
  a. Copy the stitched target images to be aligned and merged from step 60 into the ‘source’ folder, maintaining the filename format “XXXX_Combined.ome.tif”.
  b. Open the ‘channel_name’ directory.
    i. Rename files to match the corresponding input image names excluding the.ome.tif extension.
    ii. Assign channel names for every image layer.
  c. Open main.ipynb.
    i. Verify the parameter settings.
    ii. Run the program.
      ***Note:*** The aligned single image is saved in the aligned folder with filenames containing the suffix ‘al’.
  d. If output of the final merged TIFF file is enabled (default setting), the program generates two files in the same directory as main.ipynb:
    i. an uncompressed image file named final_image.ome.tif.
    ii. a compressed pyramidal image named pyr_final_image.ome.tif to enable fast visualization in QuPath.
      ***Note:*** The compressed pyramidal image is typically approximately 1.5× larger than the combined size of the stitched input images when all channels are included. For example, overlaying three images with a resolution of 2792 × 3720 pixels and a file size of 316 MB each results in a final 16-plex image with the same spatial resolution and an approximate file size of 1.4 GB.

### Image analysis

**Timing: 30 min per slide**

**Figure 6:**
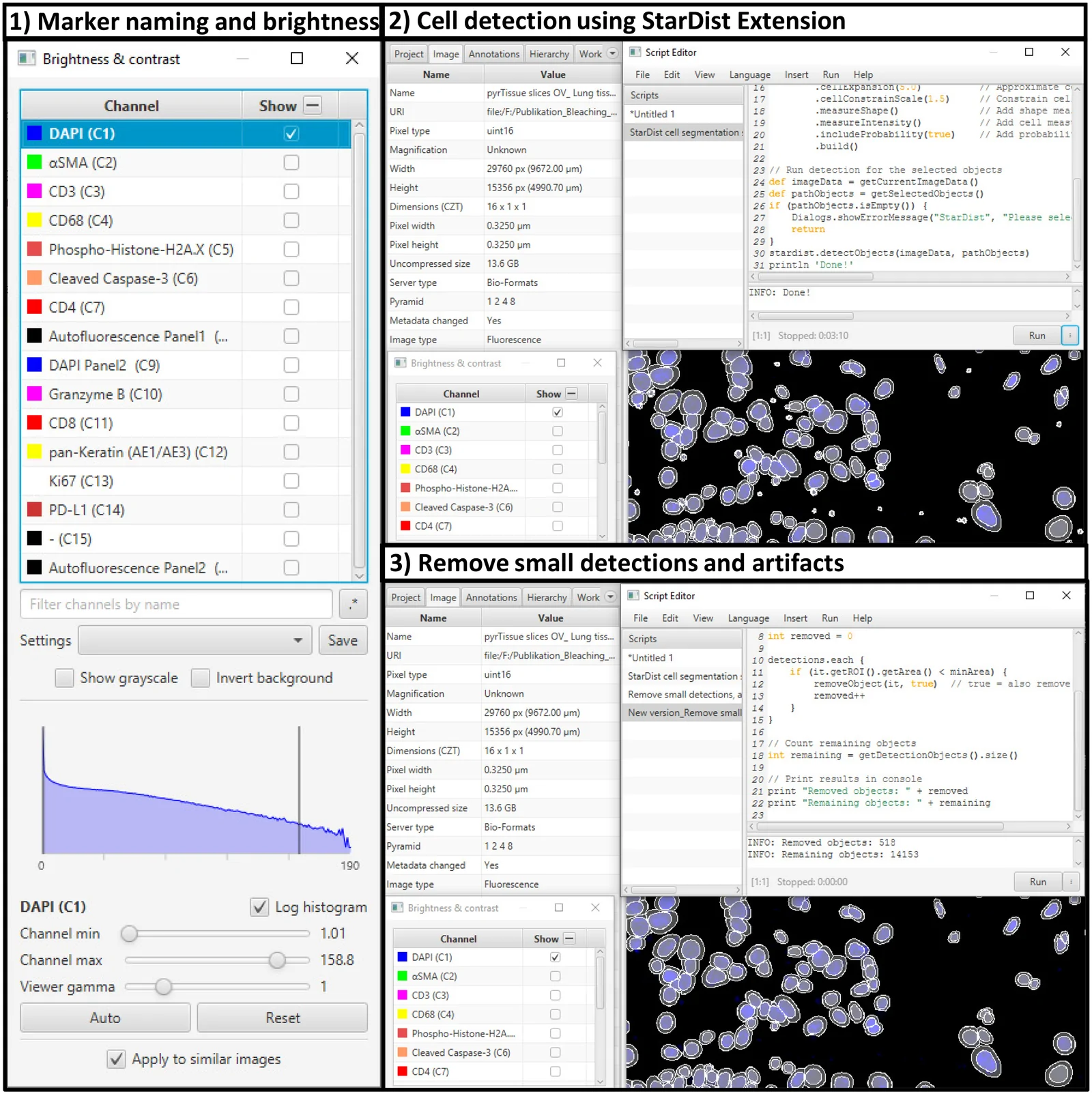
Part 1: Image analysis workflow in QuPath (1) Marker naming and brightness. Set name of each channel and adjust the values of each channel. (2) Cell detection using StarDist Extension. (3) Optional: Remove small detections and artifacts.

**Figure 7:**
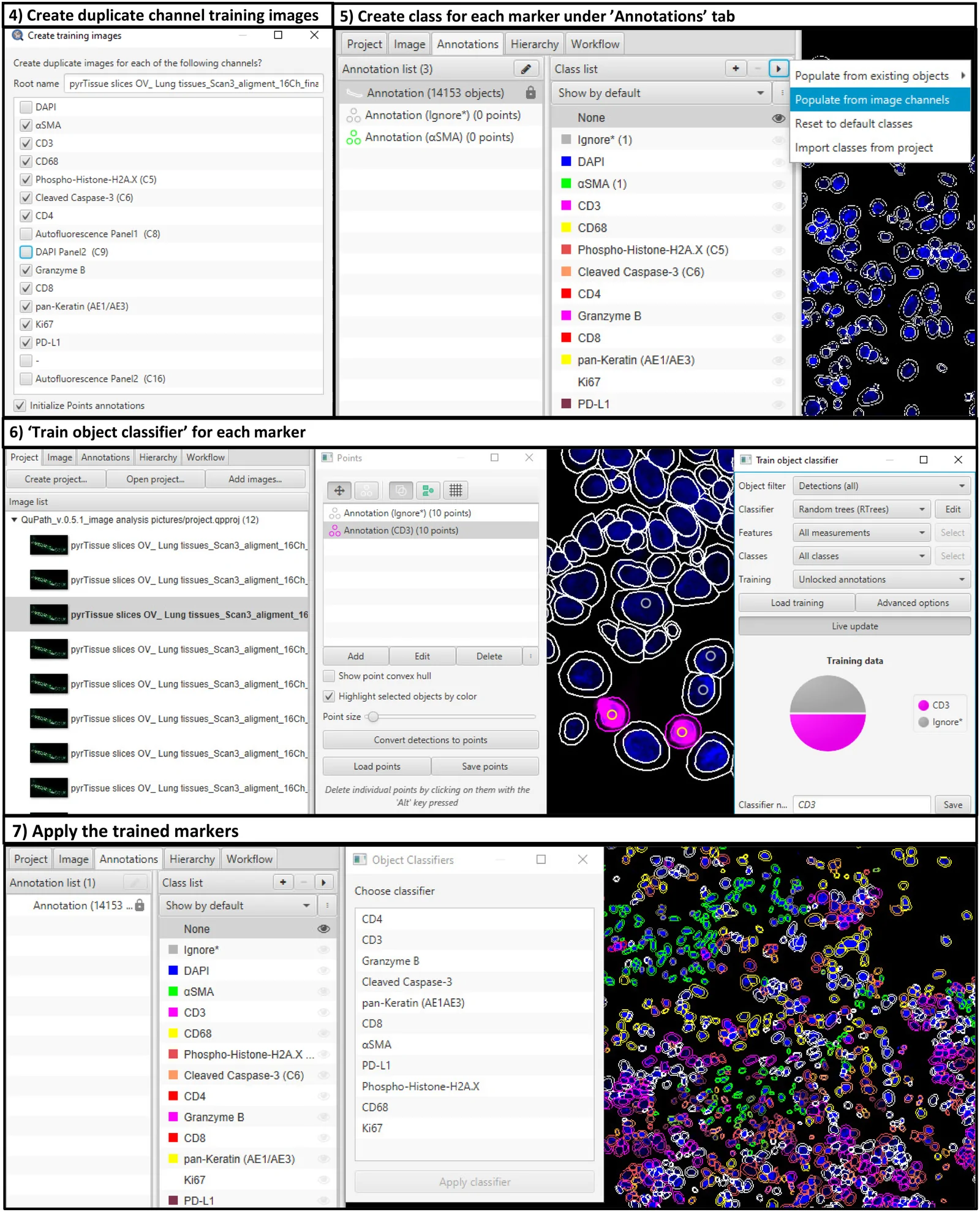
Part 2: Image analysis workflow in QuPath (4) Create duplicate channel training images. (5) Create class for each marker under ‘Annotations’ table. (6) ‘Train object classifier’ for each marker. (7) Apply the trained markers.

**Figure 8:**
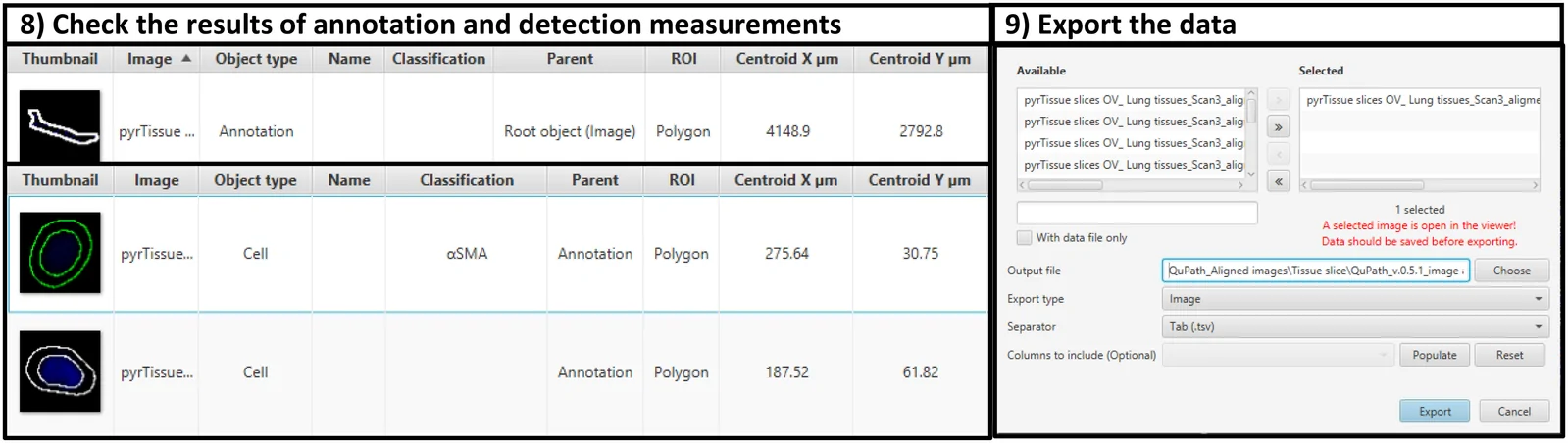
Part 3: Image analysis workflow in QuPath (8) Check the results of annotation and detection measurements. 9) Export the data.

This step describes the basics of image analysis using the open-source software QuPath, including cell recognition (Figure 6), training for the characterisation of individual markers (Figure 7), and data export (Figure 8), which opens up further analysis possibilities.

***Note:*** The protocol is compatible with various QuPath^5^ versions, with the following versions being tested: 0.4.4, 0.5.1, 0.6.0.

***Note:*** Before image analysis can be started, install the StarDist^8^ extension, as this is used for cell nuclei segmentation.

62. Open QuPath and ‘Create Project’ and ‘Add Images’ to analyze.
63. Defining the default settings (Marker naming and brightness).
  a. Press button ‘Show the brightness & contrast dialog’ (Short: Shift + C).
  b. Set name of each channel if necessary (by double clicking on channels).
  c. Adjust the values of each channel (by dragging channel min and max).
  d. Transfer the markers to the ‘Annotations’ tab: ‘Populate from image channels’.
  e. Make annotation around the tissue to analyze.
64. Cell detection.
  a. Use StarDist Extension (scripts can be found here).
    i. Check Image > Pixel width and height.
    ii. Automate > Show script editor > Choose ‘StarDist cell segmentation’ script with right pixel size and drop it to the script editor > Select annotation > Run.
    iii. Pop-up window ‘Please select your StarDist cell segmentation file’ > OK > Select ‘stardist_cell_seg_model.pb’ > Open.
    iv. Save Project.
  b. Alternative: Use QuPath cell detection: Analyze > Cell detection > Set parameters (adapt threshold until satisfied) > Run.
65. Optional: Remove small detections and artifacts (script can be found here).
  a. Automate > Show script editor > Choose ‘Remove small detections, artifacts’ script > Set minArea > Run.
  b. Adapt minimal area of detections if necessary.
  c. Save Project.
66. Train models to recognize positive marker signals.
  a. Classify > Training images > Create duplicate channel training images.
    i. Select channels you want to analyze > Tick ‘Initialize Points annotations’.
    ii. Duplicate image for each channel is created.
    iii. Create Class for each marker under ’Annotations’ tab (Short: Click ‘Triangle/Three dots’ > ‘Populate from image channels’).
  b. ‘Train object classifier’ for each marker.
    i. Choose duplicated channel marker you want to analyze by selecting the image under ‘project tab’.
    ii. Select ‘Click to add points to an annotation’ > Add two annotations if not present already.
    iii. Set Class of annotations: Marker that you train and ‘Ignore’ class.
    iv. Mark some cells for each group (similar number) > ‘Ignore’ for the negative cells and ‘Annotation (*Marker*)’ for positive cells.
    v. Classify > Object Classification > Train object classifier > Live update.
    vi. Type in marker as ‘Classifier name’ > Save.
  c. Apply the trained markers.
    i. Go back to original image in the ‘Project’ tab.
    ii. Classify > Object classification > Load object classifier > Choose classifier and apply classifiers sequentially.
67. Check the results and export the data.
  a. Measure > Show annotation/detection measurements.
  b. Export the data: Measure > Export measurements (Choose data, output direction, export type and separator).

## Expected outcomes

Based on our developed sequential bleach-and-stain multiplex workflow, a 16-plex immunofluorescence staining panel was applied to various tissue types, including tonsil, pancreatic ductal adenocarcinoma, ovarian cancer, lung cancer, mantle cell lymphoma, breast cancer and hepatocellular carcinoma. The method demonstrates both broad applicability across different tissue types and expanded marker coverage within a single tissue section.

In this study, a composite 16-plex panel for the identifcation of immune cell infiltration and cell cycle state was implemented across three cycles of Opal staining separated by chemical photobleaching steps. This enables comprehensive profiling of tumor cells, stromal components, and immune populations for detailed analyses of cellular composition, functional states, DNA damage, apoptosis, immune infiltration, and immune checkpoint marker expression.

**Figure 9:**
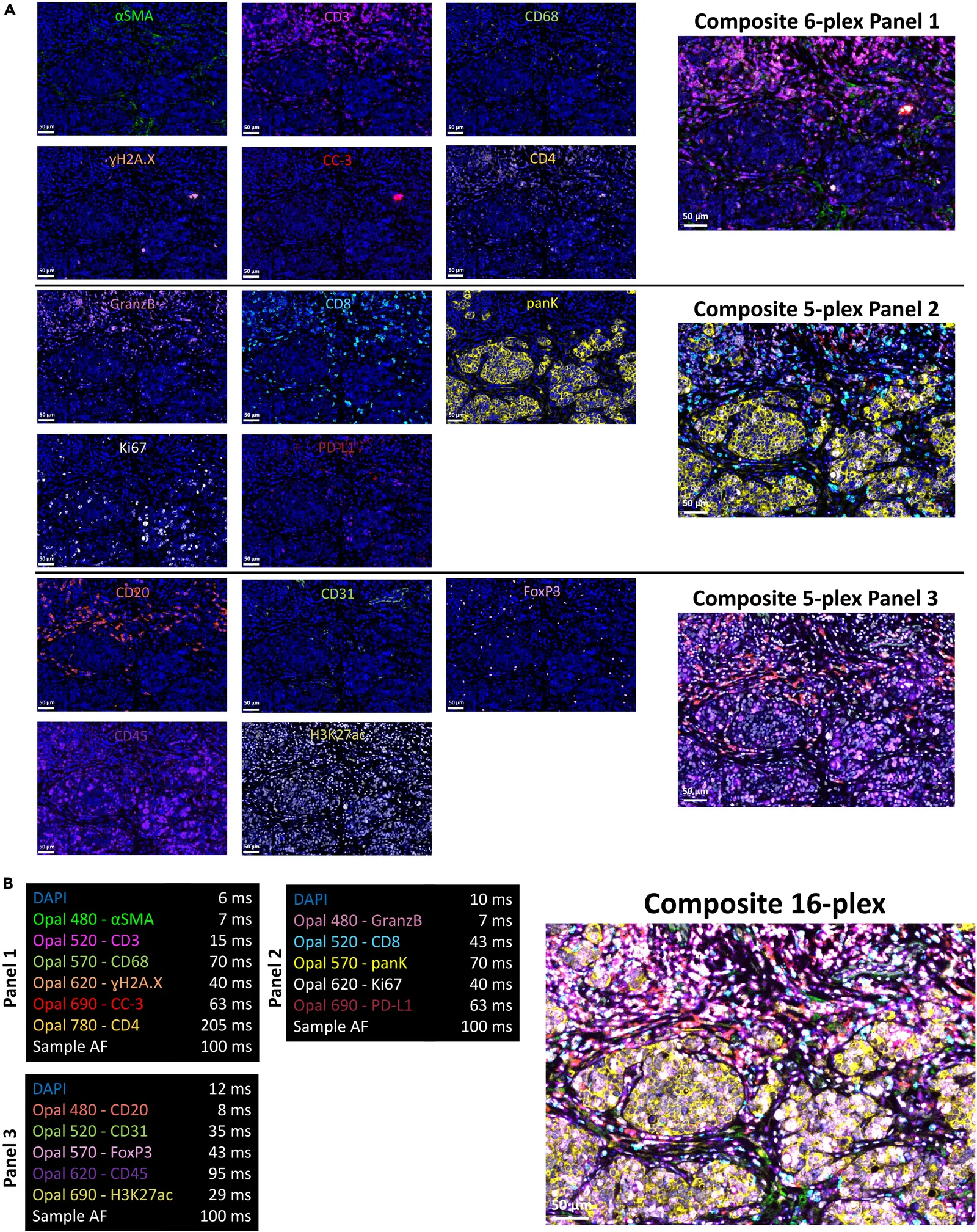
Multiplex immunofluorescence staining of an ovarian tumor section (A) Representative images of an ovarian tumor tissue section stained for 16 markers. First, a 6-plex panel was stained with αSMA, CD3, CD68, phospho-Histone-H2A.X (ɣH2A.X), Cleaved Caspase-3 (CC-3), CD4, followed by two 5-plex panels: a second panel with Granzyme B (GranzB), CD8, pan-Keratin AE1/AE3 (panK), Ki67, PD-L1 as well as a third panel with CD20, CD31, FoxP3, CD45, H3K27ac on the same tissue section. Between each staining cycle, the fluorophores were inactivated by chemical photobleaching. Shown are single-marker and composite images for Panel 1, Panel 2 and Panel 3 illustrating marker distribution within the same tissue section. Scale bars indicate 50 µm. (B) Composite image of the merged 16-plex immunofluorescence staining of the ovarian tumor section from (A) with the mentioned panels. The figure illustrates the spatial distribution of all markers within the same tissue section, along with the exposure times used for each imaging channel. Scale bar indicates 50 µm.

The marker identity, staining positions, antibody dilutions, and fluorophores are summarized in Table 1 and complemented by representative single-marker and composite images illustrating panel performance and image quality (Figure 9A). Images from three panels were unmixed, stitched, and aligned using Python, generating a single, high-resolution image containing all 16 markers with exact cell-to-cell registration (Figure 9B). Thus, accurate colocalization, cell neighborhood, and spatial interaction analyses at single-cell resolution can be performed on a single tissue section. Compared with consecutive section analysis, an exact alignment of any marker of interest is feasible, allowing true single-cell comparisons. The methodology demonstrates how chemical photobleaching-based multiplexing extends a standard 6-plex panel to 16 markers, with the possibility of adding further markers through additional bleach-and-stain cycles. The workflow is compatible with conventional TSA-based reagents and standard fluorescence imaging platforms, and can be adapted to different marker panels depending on the biological question, with the number of achievable cycles ultimately limited by tissue stability.

**Table 1:** Multiplex panel layout and antibody–fluorophore assignments

Staining round	Staining position	Target protein	Antibody dilution	Fluorophore
Cycle 1	1	αSMA	1:100	Opal 480
	2	CD3	1:100	Opal 520
	3	CD68	1:300	Opal 570
	4	phospho-Histone-H2A.X	1:75	Opal 620
	5	Cleaved Caspase-3	1:75	Opal 690
	6	CD4	1:100	Opal 780
Cycle 2	7	Granzyme B	1:50	Opal 480
	8	CD8	1:100	Opal 520
	9	pan-Keratin (AE1/AE3)	1:50	Opal 570
	10	Ki67	1:100	Opal 620
	11	PD-L1	1:100	Opal 690
Cycle 3	12	CD20	1:100	Opal 480
	13	CD31	1:800	Opal 520
	14	FoxP3	1:100	Opal 570
	15	CD45	1:100	Opal 620
	16	H3K27ac	1:400	Opal 690

Staining position, target protein, antibody dilution, and Opal fluorophore used across three staining cycles.

## Quantification and statistical analysis

Following image stitching, registration, and overlay using the Python/Jupyter Notebook workflow described in this protocol, quantitative fluorescence analysis was performed in QuPath (v0.6.0). Regions of interest (ROIs) were manually defined on representative tissue areas and applied consistently for quantitative measurements. For autofluorescence analysis, fluorescence intensity was quantified across all acquired fluorescence channels before and after chemical photobleaching. For fluorophore inactivation analysis, fluorescence intensity was quantified in the corresponding Opal fluorescence channel before and after chemical photobleaching.

For autofluorescence analysis, the median fluorescence intensity across all acquired fluorescence channels was quantified within each ROI because the median provides a robust estimate of the typical autofluorescence signal while minimizing the influence of isolated bright structures and imaging artifacts. Autofluorescence reduction was calculated as the percentage decrease relative to untreated control sections.

For fluorophore inactivation analysis, mean fluorescence intensity was quantified within each ROI in the corresponding Opal fluorescence channel to assess the overall residual Opal fluorophore signal after chemical photobleaching. Signal reduction was calculated as the percentage decrease in fluorescence intensity before and after bleaching. For time-course experiments, fluorescence intensity was expressed as the reciprocal of the normalized auto-exposure time relative to the initial measurement, providing a relative measure of the remaining fluorophore signal while maintaining image acquisition within the linear dynamic range.

Statistical analyses were performed using GraphPad Prism (v11.0.0). Differences between treatment conditions were analyzed using one-way ANOVA followed by Šídák’s multiple comparisons test, as indicated in the corresponding figure legends. Data are presented as mean ± SD, with individual ROI measurements shown where applicable. The number of ROIs and biological replicates analyzed varied depending on the experiment and are specified in the corresponding figure legends.

## Limitations

This protocol has inherent limitations that must be taken into account. Repeated HIER steps can progressively damage the tissue, leading to tissue detachment and loss of structural integrity, which limits the number of staining cycles that can be performed.

The method is time-consuming, as staining with a single fluorophore requires approximately 3 h. To increase throughput, the workflow can be adapted to an autostainer suitable for the Akoya system.

Furthermore, some proteins exhibit reduced stability or altered antigenicity after multiple HIER steps, making their detection unreliable in later cycles. Sensitive markers should therefore be placed early in the staining sequence, thus, the total number of sensitive markers used in a study is limited.

Finally, high-plex imaging generates large and computationally intensive datasets, and the registration of sequentially stained slides can exceed the capacity of standard computers, especially for large tumour tissues. While smaller samples such as biopsies, tissue microarrays, and organoids can typically be processed without difficulty, larger sections may require powerful computing resources to ensure efficient data processing and analysis.

## Troubleshooting

### Problem 1

Detachment of tissue parts.

### Potential solution

• Choice of slide: Use adhesive microscope slides (positively charged, Poly-L-Lysine coated).
• Incubation of slides: Allow slides to dry at room temperature before baking at 56 °C for a longer time period.
• pH buffer: Replace pH 9 retrieval with multiple pH 6 retrieval cycles when possible and transfer antibodies that require pH 9 to the end of staining. Reserve pH 9 for markers that strictly depend on it.
• HIER: Shorten HIER incubation or switch to different HIER methods such as microwave, water bath, or pressure cooker while maintaining effective stripping.
• Bleaching: Adjust bleaching solution by reducing H_2_O_2_ concentration, but make sure to maintain fluorophore inactivation.
• Heat generation during bleaching: Prevent heat damage during bleaching by cooling the environment or pausing to allow the lamp to cool.

### Problem 2

High background, low signal-to-noise ratio (visible during unmixing process, step 58).

### Potential solution

• Antibody concentration: Reduce antibody concentration and, if needed, increase fluorophore concentration to improve specific binding.
• Antibody-Opal pairing: Consider modifying the Antibody-Opal pairing. Some Opal fluorophores (Opal 480, 520) show higher AF due to spectral overlap with AF spectrum and should be assigned to markers with strong, clear signals.
• Unmixing library: Verify the unmixing library in inForm® Tissue Analysis Software, especially for channels prone to AF (Opal 480, 520).
• Washing steps: Extend or increase washing steps.

### Problem 3

Ineffective Bleaching.

### Potential solution

• Incubation time: Extend incubation time in bleaching solution under light exposure.
• Composition of bleaching solution: Increase concentration of H_2_O_2_ (e.g., 4.5%) or add NaOH (e.g., 20 mM), while monitoring tissue integrity.
• Light source: Bleaching efficiency depends on the light intensity and spectrum. Full-spectrum lamps generally provide the best performance, but all fluorophores should be tested to confirm effectiveness. LED lamps are preferable to halogen lamps due to higher light output and lower heat generation. Ensure that the light source directly beams the sample.
• Antibody concentration: Reduce antibody concentration to improve bleaching efficiency while maintaining detectable signal.
• Exposure time: Reduce the exposure time for less bleachable fluorophores for imaging of further staining cycles after bleaching. This prevents carryover of signals into subsequent cycles.
• Thresholding: Apply thresholding during analysis to remove background signals.

### Problem 4

Weak or inconsistent marker signals in multiplex staining (visible during unmixing process, step 58).

### Potential solution

• Antibody-Opal pairing: chose antibody-Opal assignments dependent on marker expression, abundance and colocalisation.
  ○ Pair weakly expressed markers with brighter fluorophores (Opal 480, 520, 570).
  ○ Assign strongly expressed markers to dimmer fluorophores (Opal 620, 690, 780).
  ○ Avoid assigning co-expressed markers to adjacent fluorophores or positions, as incomplete unmixing and bleed-through can introduce artifacts.
• Panel position (stick to the positioning experiment Figure 2B):
  ○ Place markers with HIER-sensitive epitopes in earlier positions.
  ○ Place markers whose epitopes are unmasked by repeated HIER steps in later positions.
• Antibody concentration and incubation:
  ○ Weak, punctate or homogeneous signal may indicate under-saturation of epitope bindings. In this case, increase primary antibody concentration or incubation time.
  ○ Overly strong signals that obscure structure suggest over-saturation. In this case, decrease primary antibody concentration.
• Opal concentration:
  ○ Increase Opal concentration (≥1:50) for weak signals.
  ○ Decrease concentration (≤1:2000) for overly strong signals.

### Problem 5

Wrong signals in adjacent channels or positions.

### Potential solution

• Antibody-Opal pairing: Avoid assigning adjacent fluorophores to co-expressed markers to prevent spectral overlap and artifacts due to incomplete unmixing.
• Position assignment: Ensure that co-expressed markers are not in adjacent positions, as incomplete stripping in HIER can cause false positives in subsequent channels.
• HIER step: Extend HIER duration or adjust the method to improve antibody stripping.
• Drop out controls: Use drop-out controls to verify that neighboring markers are effectively stripped. After staining and performing the antibody stripping process, omit the primary antibody in the next cycle and add only the secondary antibody with the Opal fluorophore. If signals from both fluorophores remain after image acquisition, the stripping was incomplete. To resolve incomplete stripping, extend the HIER step duration or switch to an alternative HIER method. A detailed description of how to perform drop-out controls can be found in the Opal Multiplex IHC Assay Development Guide.

### Problem 6

Marker paired with Opal 480 shows no signal or high background.

### Potential solution

• Incorrect unmixing: Unmixing process is incomplete or subtracts positive signals in the Opal 480 channel. Re-evaluate and adjust the unmixing settings in the inForm® Tissue Analysis software to improve the separation of fluorophore signals, or assign Opal 480 to a more abundant marker with a clear signal.

## Resource availability

### Lead contact

Further information and requests for resources and reagents should be directed to and will be fulfilled by the lead contact, Meng Dong (meng.dong@ikp-stuttgart.de).

### Technical contact

Technical questions on executing this protocol should be directed to and will be answered by the technical contacts, Elias Stahl (elias.stahl@ikp-stuttgart.de) or Yang Zhang (yang.zhang@outlook.de).

### Materials availability

This protocol did not generate new unique materials.

### Data and code availability

The source code generated for the image processing pipeline is available on GitHub at https://github.com/Zha2Git/Stitching_Alignment_Tool. A version of record corresponding to the code has been archived on Zenodo and is available at https://doi.org/10.5281/zenodo.21048347. The code can be tested with the deposited raw data at https://cloud.uni-jena.de/s/g92RdL4ejc2YGDM. Two movies to demonstrate the image unmixing as well as image stitching, alignment, and overlay are available at https://cloud.uni-jena.de/s/g92RdL4ejc2YGDM. Additional raw data are available upon request to the corresponding author.

## Acknowledgments

The research was supported by 10.13039/501100001646Robert Bosch Stiftung and 10.13039/100008672Wilhelm Sander Stiftung (no. 2024.007.1). We thank Kerstin Willecke, Isabel Ernst, and Luise Atlas for their excellent technical assistance. The graphical abstract (https://BioRender.com/u8ozd24), Figure 1 (https://BioRender.com/78m4tti), and Figure 5 (https://BioRender.com/3k5g0i7) were created using Biorender.com.

## Author contributions

Conceptualization, E.S., Y.Z., W.S., and M.D.; investigation, E.S., Y.Z., J.A.S., J.T., and C.L.; writing – review and editing, E.S., Y.Z., W.S., and M.D.; funding acquisition, W.S. and M.D.; supervision, W.S. and M.D.

## Declaration of interests

The authors declare no competing interests.

## Declaration of generative AI and AI-assisted technologies in the writing process

During the preparation of this work, the authors used ChatGPT for assist with language editing and text polishing. The authors reviewed and edited the output as needed and take full responsibility for the content of the published article.
